# The role of natural gas as a primary fuel in the near future, including comparisons of acquisition, transmission and waste handling costs of as with competitive alternatives

**DOI:** 10.1186/1752-153X-6-S1-S4

**Published:** 2012-04-23

**Authors:** Fang-Yu Liang, Marta Ryvak, Sara Sayeed, Nick Zhao

**Affiliations:** 1 University of Chicago, Chicago, IL, USA

## Abstract

Natural gas comprises about a quarter of the United States’ energy use. It is more environmentally friendly than oil and coal due to lower carbon dioxide (CO_2_) emissions per unit, less costly per unit of energy and more readily available domestically in abundant supply. However, due to a number of barriers in the political, infrastructural, pricing and other arenas, the use of natural gas as a significant energy source in the United States has been limited. In our paper, we highlight the favorable qualities of natural gas and its benefits for the consumer, producer, and environment, having compared the costs of the various components of the natural gas business such as drilling and transport to that of coal and oil. Moreover, we touch upon the major issues that have prevented a more prevalent use of the gas, such as the fact that the infrastructure of natural gas is more costly since it is transported though pipelines whereas other energy sources such as oil and coal have flexible systems that use trains, trucks and ships. In addition, the powerful lobbies of the coal and oil businesses, along with the inertia in the congress to pass a national climate change bill further dampens incentives for these industries to invest in natural gas, despite its various attractive qualities. We also include discussions of policy proposals to incentive greater use of natural gas in the future.

## Introduction

Natural gas is formed in the earth’s crust as a result of transformation of organic matter due to heat and pressure of overlying rock. The gas hydrocarbons can also be produced as a result of microbial decomposition of organic substances and due to reduction of mineral salts [1]. Some of these gases are released into the atmosphere or hydrosphere while the rest accumulates in the upper layers of the earth’s crust.

The composition of natural gas varies depending on a number of factors like the origin, location of deposit and geological structure. Natural gas mainly consists of saturated aliphatic hydrocarbons like methane. Components such as carbon dioxide, hydrogen sulfide, nitrogen and helium constitute an insignificant proportion of natural gas composition. Natural gas is the cleanest of all fossil fuels and the main products of combustion of natural gas are carbon dioxide and water vapor. The combustion of natural gas releases very small amounts of nitrogen oxides (NOx), sulfur dioxide (SO_2_), carbon dioxide (CO_2)_, carbon monoxide (CO), other reactive hydrocarbons and virtually no particulate matter. Coal and oil are composed of much more complex molecules and when combusted, they release higher levels of harmful emissions such as nitrogen oxides and sulfur dioxide. They also release ash particles into the environment. Table 1 summarizes the different chemical emissions of competition fuels.

**Table 1:** Comparing the GHG emissions of several fossil fuels

Pollutant (pounds per billion btu of energy input)	Natural gas	Oil	Coal
Carbon dioxide	117,000	164,000	208,000
Carbon monoxide	40	33	208
Nitrogen oxides	92	448	457
Sulfur dioxide	1	1,122	2,591
Particulates	7	84	2,744
Mercury	0.000	0.007	0.016

Source: EIA - Natural Gas Issues and Trends 1998

Natural gas can be used in many ways to help reduce the emissions of pollutants into the atmosphere as it emits fewer harmful pollutants and an increased reliance on natural gas can potentially reduce the emission of many of these harmful pollutants. In the United States the pollutants emitted from the combustion of fossil fuels have led to the development of a number of pressing environmental problems that include, but is not limited to:

• Emission of greenhouse gases, which could contribute to global warming

• Smog, air quality and acid rain, which is detrimental to human health and the wider ecosystem

Global warming is an environmental issue that deals with the potential for global climate changes due the increased levels of atmospheric greenhouse gases. Scientists claim that an increase in greenhouse gases will lead to increased temperature around the globe. The principle greenhouse gases include carbon dioxide, water vapor, methane and nitrogen oxides. The levels of greenhouse gases in the atmosphere have been increasing due to the widespread burning of fossil fuels by the growing human populations.

The main component of natural gas, methane, is itself a potent greenhouse gas. Methane emissions account for only 1.1% of the total U.S. greenhouse gas emissions, they account for 8.5% of the greenhouse gas emissions based on global warming potential. A study performed by the EPA (Environment Protection Agency) and the GRI (Gas Research Institute) in 1997 lead to the conclusion that the reduction of emissions from increased natural gas use would strongly outweigh the detrimental effects of increased methane emissions [2]. Therefore the increased use of natural gas can serve to reduce the emission of greenhouse gases in the United States.

Smog is formed by a chemical reaction of carbon monoxide, nitrogen oxides, volatile organic compounds and heat from sunlight. Ground level ozone and smog can contribute to respiratory problems that range from temporary discomfort to permanent lung damage. The use of natural gas does not contribute to the formation of smog as it emits low levels of nitrogen oxides and no particulate matter. Increased natural gas use could be served to combat smog production. This would reduce the emissions of smog causing chemicals and result in healthier air.

Acid rain is formed when sulfur dioxide and nitrogen oxides react with water vapor and other chemical in the presence of sunlight. The increased use of natural gas could provide for fewer acid rain causing emissions.

Natural gas powered industrial application and natural gas fired electric generation offer a variety of environmental benefits and environmentally friendly uses that include [3]:

**1) Fewer GHG emissions** [see Table 1]

**2) Re-burning**: Natural gas can be added to coal or oil fired boilers to reduce NOx and SO_2_ emissions.

**3) Reduced sludge**: Sludge refers to the residual material left from industrial waste water, or sewage treatment processes. Coal-fired power plants and industrial boilers that use scrubbers to reduce SO_2_ emission levels usually generate thousands of tons of harmful sludge. Natural gas releases insignificant amounts of SO_2_, which eliminates the need for scrubbers, and thus reducing the amount of sludge from industrial processes.

**4) Cogenerations**: Cogeneration is the use of a heat engine or a power station to simultaneously generate both electricity and useful heat. The preferred fuel for new cogeneration equipment is natural gas.

**5) Fuel cells** [see Additional file 1]

**6) Combined cycle generation** [See next section]

## Use of natural gas

Natural gas has a number of applications commercially in homes, industries and the transportation sector.

### Residential use

Natural gas is one of the cheapest forms of energy available to residential consumers; it is even cheaper than electricity as a source of energy. According to Department of Energy, in 2007 natural gas was the lowest cost conventional energy source available for energy use; it costs less than 30% the cost of electricity, per Btu (British thermal unit). [See Table 2 for the exact costs]

**Table 2:** Installing residential natural gas distribution

Energy source	Residential energy costs per Btu
**Kerosene**	$28.81
**Propane**	$27.70
**No. 2 heating oil**	$24.56
**Natural gas**	$11.01
**Electricity**	$34.14

Source: Duke Energy Gas Transmission Canada 2011

Natural gas is used for heating and cooking. Cooking with natural gas provides benefits like easy temperature control, self-ignition and self-cleaning. A gas range costs about half that of an electric range and it has quick heat ability. The newer generation natural gas ranges are most efficient, economical and versatile cooking appliances.

Natural gas is the most popular fuel for residential heating. According to American Gas Association (AGA), 51% of the heated homes in US used natural gas heating in 2000.

Natural gas air conditioning units, like many other gas powered appliances are initially more expensive than electric ones but are cheaper to operate and they have longer expected life and require low maintenance. Because natural gas requires very little electricity, it frees up electric service in existing buildings for other applications. Not only are electric service needs in new facilities can be dramatically reduced, less electric demand also means less requirement and expense for emergency back-up generation [4]. Modern residential air conditioner units use close to 30 percent less energy than in years past, and have an expected working life of 20 years with very little maintenance [5]. All gas-powered appliances offer a safe, efficient, and economical alternative to other fuel sources. Almost 70% of the new homes in USA use natural gas for heating and therefore a large number of them already have natural gas delivery infrastructure in place. Gas pipes that can supply gas to furnaces can be used to supply energy for all gas-powered appliances, thus installation is easy.

Natural gas fuel cells and micro turbines offer residential consumers the capacity to disconnect from their local electric distributor, and generate just enough electricity to meet their requirements. Although this technology is still in its early stages, it promises to provide independent, efficient, reliable and environmentally friendly electricity for residential needs.

### Commercial uses

The main commercial uses of natural gas include space heating, water heating and cooling. See Table 3 for the exact percentage allocated to each type of use. According to the Energy Information Administration, as of the year 2003, the commercial sector consumed about 6,523 trillion Btu of energy annually (minus electrical system losses) most of which was used for heating, lighting and cooling purposes.

**Table 3:** Commercial uses of natural gas

Commercial energy use	Percentage
**Space heating**	36%
**Cooling**	8%
**Ventilation**	7%
**Water heating**	8%
**Lighting**	20%
**Other**	21%

Source: Washington Policy and Analysis Inc, Fueling the Future 2000

Natural gas is an efficient and economical fuel for commercial buildings. Non-space heating applications of natural gas are expected to account for the majority of the growth of natural gas use in the commercial sector. It provides 13% of the energy used in commercial cooling but this percentage is expected to increase due to technological innovations in commercial natural gas cooling techniques.

In particular, there has been a growth in the demand for natural gas in the food service industry. Natural gas is a flexible energy source and natural gas–powered appliances can cook food in many different ways that are economical and efficient for large commercial food preparation establishments. Smaller systems that use natural gas can integrate gas-fired fryer, griddle, over, hot/cold storage areas and multiple venting options in a small space as natural gas-powered appliances can be easy and efficient while being compact.

Technological advancements allow natural gas to be used to increase energy efficiency in commercial settings. Natural gas-powered fuel cells, reciprocating engines and turbines can generate electricity. These natural gas powered units offer commercial environments more independence from power disruption, along with consistent high-quality electricity and control over their own energy supply.

Moreover, combined heating and power (CHP) and combined cooling, heating and power (CCHP) systems are used to increase energy efficiency. These systems are able to use energy that is normally lost in the form of heat and by using this energy that is normally wasted, energy efficiency can be dramatically improved. For example, in a certain industrial setting, the excess heat and steam produced by this process may be used to fulfill other industrial applications such as space heating, water heating and to power industrial boilers. Increased efficiency saves money and the burning attributes of natural gas helps industries reduce harmful emissions.

### Industrial uses

Natural gas helps provide base ingredients for products like plastic, fertilizer, anti-freeze and fabrics. Industry accounts for about 25% of natural gas use across all sectors. It is the second most used energy source in industry after electricity.

Natural gas is used primarily in the metal, chemical, petroleum refining, stone, clay and glass, pulp and paper, plastic and food-processing industries. These businesses account for more than 84% of the total industrial natural gas use. Natural gas is used for waste treatment and incineration, metal preheating, glass melting, drying and dehumidification, food processing and fueling industrial boilers. It is also used as feedstock for the manufacturing of a number of chemicals and products and as a building block for methanol, which has a number of industrial applications. Natural gas is converted to synthesis gas (a mixture of hydrogen and carbon oxides formed by the process of steam reforming. In the process, natural gas is exposed to a catalyst that causes oxidization of natural gas when brought in contact with steam). Synthesis gas is used to make methanol (can be used as fuel source in fuel cell) – used to make substances like formaldehyde, an additive for cleaner burning gasoline called MTBE (methyl tertiary butyl ether,) and acetic acid. Gases like butane, propane and ethane can be extracted from natural gas and these may be used as feedstock for products like fertilizers and pharmaceutical products.

Natural gas desiccant systems (used for dehumidification) are used in pharmaceutical, plastic, candy and recycling industries. The absorption systems used to heat and cool water in an economical, efficient and environmentally sound way.

### Natural gas in the transportation sector

According to natural gas vehicle coalition estimates there are 120,000 Natural Gas Vehicles (NGV) in USA and more than 8.7million NGV worldwide. There are about 1,100 natural gas fueling stations in USA alone.

Disadvantages of NGV like limited range, trunk space, higher initial cost, lack of refueling infrastructure are impediments to future spread of NGV. Some natural gas vehicles are bi-fuel [6], so there is flexibility of fuel choice. Many of these vehicles were originally just gasoline but have been converted to be bi-fuel. Conversion is costly and results in less efficient use of natural gas.

Newer, strict federal and state emission laws require an improvement in vehicle emissions, and natural gas is the cleanest burning alternative transportation fuel available and it offers the opportunity to meet the strict environmental emission standards. Natural gas is safe and lighter than air so when there is an accident, natural gas just dissipates into the air and does not form a dangerous flammable pool on the ground like other fuels. There is no pollution of ground water in the event of a spill. The natural gas storage tanks on NGV happen to be stronger and sturdier than gasoline tanks. In all, natural gas is an economical alternative to other transportation fuels. NGV are about 30% cheaper than gasoline vehicles to refuel and maintenance costs lower. Furthermore, the U.S. Environmental Protection Agency (EPA) and the National Highway Traffic Safety Administration (NHTSA) are in the process of introducing a new generation of clean vehicles through GHG emission reduction (250 million metric tons), increased energy independence (cut oil by 500 million barrels) and improved fuel efficiency from on-road vehicles and engines. This new proposal would increase the competitiveness of NGV’s by making gasoline fueled vehicles more costly.

Furthermore, natural gas use reduces environmentally harmful emissions associated with automobiles. Vehicles on the road account for 60% of the carbon monoxide pollution, 31% of the nitrogen oxides and 29% of the hydrocarbon emissions in USA. These emissions contribute to smog pollution and increase dangerous ground level ozone. Vehicles account for over half of all dangerous air pollutants and about 30% of the total carbon emissions in the USA. This contributes to the presence of greenhouse gases in the atmosphere. The environmental effects of NGV are less detrimental than that of others.

Due to the chemical composition of natural gas, NGV are much cleaner burning than others. Natural gas – methane mainly – emits small amounts of ethane, propane and butane. Gasoline/diesel fuels – contain harmful compounds – emit sulfur dioxide and nitrogen oxides (combine in atmosphere to produce ground level ozone), arsenic, benzene, nickel and over 40 other toxic substances. NGV produce, on average, 70% less carbon monoxide, 80% less nitrogen oxides and 87% less non-methane organic gas than other vehicles.

Now that the different users of natural gas have been explained, we will give examples of some technological advancement in natural gas that enhances its competitiveness.

### Infrared heating units

Natural gas is used for infrared (IR) heating units and it is an innovative and economic method of using natural gas to generate heat. IR heating units increase efficiency of powder-coating manufacturing processes. IR heaters heat materials more efficiently and quickly. Natural gas reacts with the panel of ceramic fibers containing a platinum catalyst causing a reaction with oxygen that dramatically increases temperature without producing flame. Using natural gas in this manner increases the speed of manufacturing process and it is an economic alternative to electricity.

### Direct contact water heaters

Energy from combustion of natural gas is directly transferred from the flame into the water. It is an efficient application for water heating. While normal industrial water heaters operate at 60-70% energy efficiency range, direct contact water heaters can achieve an efficiency of up to 99.7%. Therefore, there are cost savings in industries where water heating is required.

### Industrial combined heat and power

Industrial consumers get great benefits from operating natural gas Combined Cooling, Heat and Power (CCHP) systems and Combined Heat and Power (CHP) systems. Natural gas may be used to generate electricity in a certain industrial setting, the excess heat and steam produced by this process may be used to fulfill other industrial applications such as space heating, water heating and to power industrial boilers. Increased efficiency saves money and the burning attributes of natural gas helps industries reduce harmful emissions.

### Industrial co-firing

Natural gas co-firing technologies help increase industrial energy efficiency and reduce harmful emissions into the atmosphere. Co-firing is a process where natural gas is used as a supplemental fuel in combustion of other fuels like coal, biomass energy and wood. Using natural gas can improve the operational performance of the boiler including its energy efficiency. Co-firing can be used to generate electricity.

### Electricity generation using natural gas

In 2009, 23,475MW of new generation capacity are planned in the USA, out of which, 50% (12,334MW) will be natural gas fired additions. Coal is the cheapest fossil fuel for generating electricity, but at the same time it is the dirtiest. It releases the highest levels of pollutants into the air. The electricity generation industry happens to be one of the most polluting industries in the USA. New technology allows, cleaner generation of electricity using natural gas.

Natural gas is used in steam generation units, centralized gas turbines, combined cycle units, locomotives, distributed generation, industrial natural gas fired turbines, micro turbines and fuel cells.

**Appendix: electricity generation** in Additional file 1 will illustrate the market share of various fuels in electricity generation in the U.S. and the returns associated with each fuel, including nuclear, wind and coal. As demonstrated, wind, nuclear and hydroelectric power plants have the highest energy return on energy invested, but are probably less attractive due to their high fixed costs (which include construction of infrastructure, operations, maintenance, and money that needs to be spent regardless of whether power is actually produced). According to data provided by the Northwest's largest utility, PacifiCorp, the highest cost for natural cost is the “variable cost”, which could vary depending on factors like the type of technological advancements in natural gas or pollution reducing/mitigating legislation. The uncertain nature of the latter, which we should come back to later, adds to the “cost” of natural gas. Moreover, in a study presented to the Congress (also available in additional file 1), cost projections are given for each type of fuel technology, in the scenario that there is a price levied on emissions like SO_2_, NOx and CO_2_. In that hypothetical situation, natural gas combined cycle plant without carbon capture comes out as relatively competitive due to its low capital cost, high capacity factor and low emissions of CO_2_ per megawatt-hour of power generated.

With the science of natural gas explained, in the following section we will explore the natural gas markets, as this is a decisive factor in the prevalence of natural gas as a primary fuel in the future.

## Background: the pricing of natural gas

Before the 1980s, the price of natural gas generally rose and fell with oil prices. There are a number of reasons for this. One of them being that gas contracts used to be negotiated with links to crude oil or oil products, especially in International Energy Agency (IEA) [7] regions. Since then however, both oil and gas markets have been driving towards liberalization and the unbundling of network assets [8]. An instance of this is the introduction of futures contract for natural gas in the 1990 by the New York Mercantile Exchange (NYMEX), which is made possible by the creation of an efficient way to coordinate multiple players in the gas industry by delivering accurate up-to-date information on the price of natural gas, called “hub pricing”. This essentially makes natural gas into a tradeable commodity, very much in the same way as wheat, silver and frozen pork bellies. Both physical trading and derivatives trading occur for natural gas, with the latter dominating by 10 to 12 times the value of the former [9].

The spot market for natural gas is especially important to: 1) pipeline companies that set up trading facilities in order to market their capacity services and gain higher utilization factors for their pipelines and 2) infrastructure builders who uses the prices at two points in the system as an indicator for the need for new pipeline infrastructure. Similar to other commodities, the price of natural gas is inherently volatile. Since price is a reflection of market supply and demand, the volatility of natural gas hub prices serves as a good indicator for an investor who is making investment decisions, e.g. when it is a good time as a good indicator for more investment in natural gas storage.

The hub system is becoming increasingly prevalent in North America and gas is now traded at over 40 principal centers across the North American continent. The best known is the Henry Hub in Louisiana, which is the reference point of gas for the NYMEX gas future contract. Such innovative financial instruments allow the development of a spot market for natural gas, which ensured that gas price reflect underlying issues of demand and supply, such as the availability of power plants, hydro levels, gas storage levels, oil prices, pipelines, temperature, level of industrial or commercial demand); and not just “track” that of oil prices, as was the case before. Nonetheless, this does not mean that gas pricing cannot track oil prices, for to the extent that they are substitutable; the price of one will definitely have the effect on the price of another. It should be noted however, that while gas prices have been decontrolled at the wellhead and at the bulk or wholesale level, the prices of transportation and storage services (which makes up a large part of the end user prices) remains, on the large part, regulated but the government. Whether the deregulation of such services is desirable is subject to debate. [See **Appendix: Deregulation** in Additional file 1]

**Figure 1:**
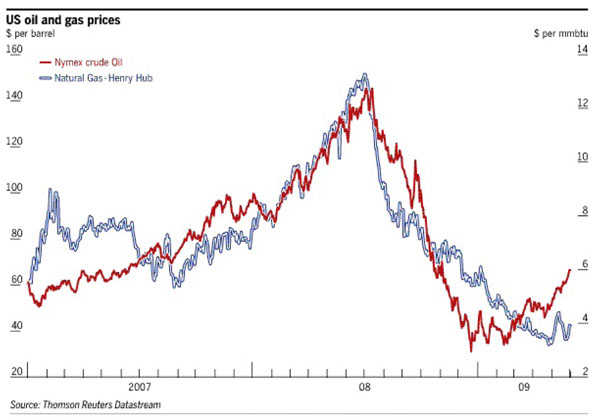
The relationship between oil and gas prices.

An interesting aspect about Figure 1 is that at some points it appears as if gas and oil prices are inversely related. Before 2007, a dip in crude oil prices is accompanied by a rise peaking in natural gas prices, the reverse is true once we hit 2007, and we witness familiar dips and peaks at times through 2008-2009. This inverse relationship in price is indicative of the high competitiveness of natural gas relative to oil (that the rise in price of one would lead to the dip in the price of another due to fuel switching).

## Gas price trends in North America

**Figure 2:**
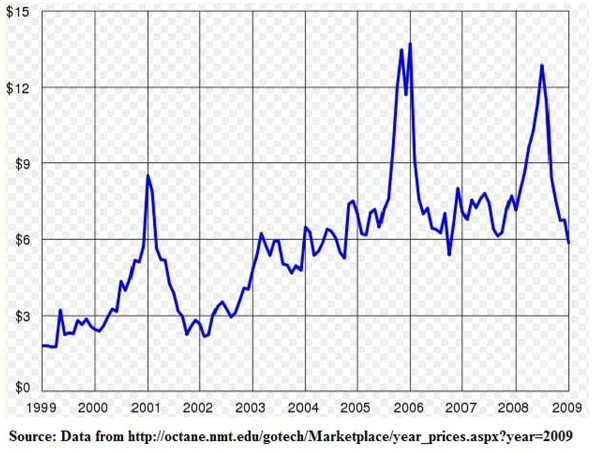
Monthly average Henry Hub prices. NYMEX, 1999-2009 (by January 08)

Ideally, liberalization is supposed to lower prices/costs and allow consumers better access to resources. However, in the case of North America, prices have been gradually increasing since the 2000s. Not only so, there has also been increased volatility in natural gas prices (the historical price volatility of natural gas has always been 20% higher than that of crude oil). [See Figure 2 for the monthly average Henry Hub prices from 1999-2009]

To understand this combined phenomenon, one should first understand the underlying powers of supply and demand of natural gas.

Common forces of demand and supply asides, there are some inherent features unique to the natural gas market that influences the structure of its price:

1) Prices of natural gas generally track the wellhead price (price of the natural gas itself as a commodity), long distance transportation cost and local distribution cost;

2) The large economies of scale associated with pipeline transmission system tend toward a natural monopoly. In this sense, a spot market for natural gas prices may not be favorable as it can stifle long term contracting, hence investments;

3) There is no exclusive end-user market in which natural gas dominates. Instead, it competes in every main use with other fuels, be it power generation, domestic heating, industrial, petrochemical etc;

4) The natural gas market in each region is influenced by the structure of entire energy market complex in each region, and by existing gas infrastructure and potential prospects for its future expansion;

5) Natural gas usually has large fixed to variable costs (fixed cost being start up capital, infrastructure etc and variable being transmission and associated costs). This usually necessitates longer term contracts in order to shift some of the large fixed costs onto consumers;

6) Indeed, balance needs to be found to smoothen the tension between the high fixed cost and low average marginal costs in order to ensure short term competitiveness without jeopardizing long term investment incentives;

7) The demand for natural gas generally has low income elasticity;

8) Different end users of natural gas have different elasticities of demand- the residential/commercial consumers are generally the least responsive to a change in price due to their inability to access viable substitutes.

Point 2) is especially important because unlike oil, gas is an infrastructure driven industry. This means that its price depends on a series of regional markets in which separate developments depend on the nature of infrastructure and regulations in place. For this reason, gas suppliers are more exposed to the risk of disruption than oil producers. In the case of oil, temporary supply shortages can be dealt with by “transporting” oil (by tanker, truck, plane or railroad) to the emergency region. On the other hand, gas requires fixed installations that are highly costly and cannot be constructed in a hurry. As a result, all gas users that are not located in the immediate vicinity of a gas field, a pipeline hub or a LNG terminal will face a dangerous risk of not being to access gas. This is a competitive drawback that cannot be ignored.

However, the extent to which such temporary supply shocks are “dangerous” should not be exaggerated, for different users have different supply needs. For example, large industrial users usually have the ability to substitute heating oil or coal, so they do not need a contract that protects them entirely from supply shocks. On the contrary, they might even prefer contracts that do not protect them from the supply shocks because they will not need to pay the “risk premium”. On the other hand, power generators with shifting requirements need greater security in supply, but the most venerable group is still the residential/public service/commercial consumers without substitution ability. Indeed, commercial consumers generally pay higher prices to enjoy protection through statutory distributor storage requirements and priority services.

One can argue that the supply risk associated with natural gas does not actually prevent it from achieving an efficient outcome. The people who need more supply security will just end up pay more. In the long run however, as oil becomes increasingly scarce and we look to natural gas as a viable option, we should aim to make natural gas more competitive in the commercial sector by reducing the supply risks, and thus the premiums paid by the consumers.

## US demand of natural gas

The North American gas market [10] is the largest in the world with 773 bcm (billion cubic meters) consumed in 2001, or 29% of global gas demand. US gas demand has been steadily rising from 1980s. Domestically, natural gas currently accounts for almost a quarter of all energy in the U.S. It heats 50% of existing home and nearly 70% of newly built homes. Gas fired power plants makes up 88% of total new electrical power plants.

**Figure 3:**
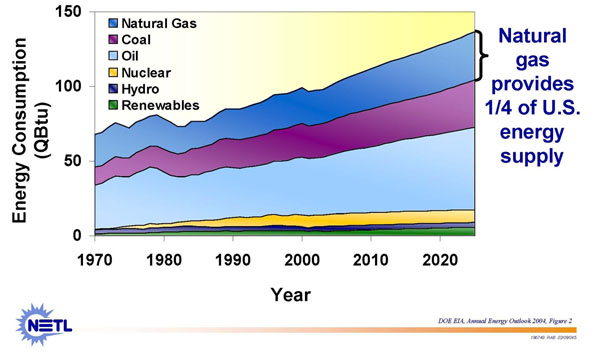
US energy consumption. Energy consumed by fuel, 1970-2025 (historical and projected)

In 2009, the EIA Annual Energy Outlook predicted that natural gas demand in the United States could reach 24.36 Tcf by the year 2030. This is a 6% increase from 2007, as compared to an expected total energy consumption increase (from all sources) of 12% (from 101.89 quadrillion British thermal units to 113.56 by 2030). The EIA predicts an annual demand increase of 0.5 percent over the next 21 years (see Figure 3). A lot of it also hinges on the prospects of a comprehensive climate change legislation. The higher the prospects, the greater the demand for natural gas as a low-carbon fuel.

In the same report, the EIA also estimates industrial energy demand to increase at an average rate of 1.2% per year to 2025 and demand for electricity to increase by an average rate of 1.8% per year through to 2025. Both are decisive to the demand of natural gas, as industrial plants and electricity generators are growing more and more concerned about energy efficiency, and promoting the idea of carbon neutrality [11]. Large utilities like AEP for example, are in the process of constructing a natural gas-fired power plant in Dresden Ohio. Similarly, Baxter International are also engaging in many energy-related GHG-reduction activities, which includes the use of innovative technologies, for which fuel switching from oil to natural gas and cogeneration are but a few examples. While some companies may be driven to energy efficiency and carbon friendly practices by profit motives, there is also external pressure coming from investors who want to see these companies engaging in sustainable practices. The Carbon Disclosure Project, for example, is non-profit organization acting on behalf of 551 institutional investors holding $71 trillion in assets under management and some 60 purchasing organizations like Dell, PepsiCo and Walmart. The CDP urges major companies to disclose their greenhouse gas emissions, water management and climate change strategies make this information available to the public, creating a major incentive for these companies to engage in environmentally friendly activities.

There has also been a proliferation of studies supporting the case that companies’ business performances are actually affected by the adoption of sustainable strategies, i.e. terms under Corporate Social Responsibility (CSR) [12]. Currently, successful businesses are starting to be defined by their integration of concepts such as management quality, environmental management, brand reputation, customer loyalty, corporate ethics and talent retention [13]. In fact, the Dow Jones Sustainability Index (DJSI) [14] was created precisely to reflect these qualities and is being increasing used by investors to evaluate companies. Such social trends are favorable to the demand of natural gas.

## Factors influencing short term v.s. long term gas demand

The short-term demand, and consequently the price fluctuations, of natural gas in the U.S. are generally cyclical/seasonal. The demand of gas generally increases during winter times because of the increased need for residential and commercial heating. This causes natural gas prices to spike in Jan/Feb and dip in July/Aug. The ratio of prices is about 2:1, and the ratio is even larger at 7.4:1when one only considers the residential sector. Base load storage capacity is designed to meet this cyclical demand. During the cold months, gas is drawn from the storage and during the hot ones, gas is stored. Unfortunately, anomalies in the weather, such as abnormally cold winters, could break down this system and cause record price volatilities. Demand shocks could also occur during times of fuel switching. Many electric generates may switch form using natural gas to using cheaper coal, thereby decreasing demand. Of course, the spot price of natural gas is also intricately tied with that of GHG emission credits, e.g. nitrogen oxide (NOx). Specifically, one would expect the price of natural gas to rise with the price of NOx, as an increase in NOx price increases the cost of NOx intensive fuels such as coal and oil.

**Figure 4:**
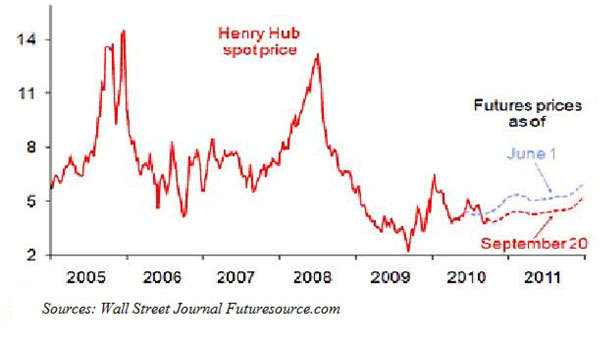
Henry Hub spot prices 2005-2001. Dollars per millions of Britisch thermal units (MMBtu).

Economy-wide recessions could also cause a demand shock, such as the recent Financial Crisis, which caused the Henry Hub spot price plunged close to $2 per MMbtu, comparable to natural gas prices in the early 1980s [see Figure 4].

Other exogenous events could also significantly impact the price of natural gas. One example is the California Energy Crisis, also known as the Western U.S. Energy Crisis of 2001, which caused the price of natural gas in California to be artificially inflated.

Long term determinants of demand are more decisive to the future role of natural gas. These include the prospects of climate change legislation in the U.S., which is discussed later. Changing demographics could also play a role. According to the Work Progress Administration, recent demographic trend have tended towards warmer Southern and Western states, which could increase demand for cooling. Other factors include energy efficiency regulations and technological advancements [See **Appendix: Technology** in additional file 1].

## Demand response to changes in natural gas prices

Conventional economic theory tells us that in the long run, consumers will react to prices and what remains for us is explore is whether this happens in the market for natural gas. As energy prices have risen steadily over the past couple of years, there has been an observed tendency to lower ammonia and methanol output and switch production to sites near cheap sources of gas. However, since the North American prices are not indexed, a supply shock/price spike will decrease consumption, regardless of whether the consumer buy spot prices or fixed price gas. In the case of the latter the consumer will just stop buying if the sport price becomes too high, in the case of the former the consumers might have to sell back to the market for a profit and thereby interrupting his/her own consumption.

## Supply in the US market

**Figure 5:**
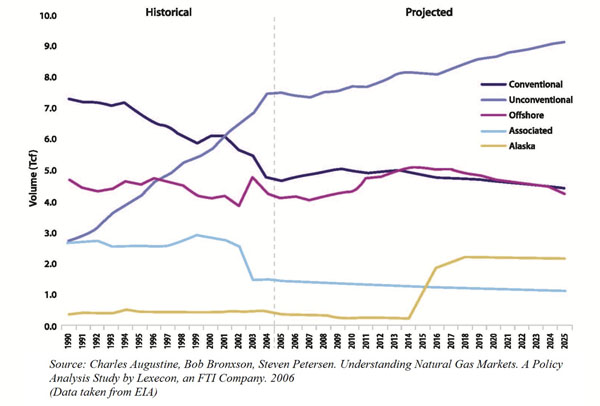
US natural gas production by source, 1990-2025.

The United States has the biggest gas market in world. Proven gas reserves amounted to 7.8 Tcm at the beginning of 2002, which is 4% of world gas reserves [See Figure 5]. Table 4 shows EIA estimates of natural gas reserves in the United States.

**Table 4:** Technically recoverable natural gas reserves in the United States

Natural gas resource category (trillion cubic feet)	As of January 1, 2007

**Non-associated gas**	
**Undiscovered**	**373.20**
Onshore	113.61
Offshore	259.59
**Inferred reserves**	**220.14**
Onshore	171.05
Offshore	49.09
**Unconventional gas recovery**	**644.92**
Tight gas	309.58
Shale gas	267.26
Coalbed methane	68.09
**Associated-dissolved gas**	**128.69**
**Total lower 48 unproved**	**1366.96**
**Alaska**	**169.43**
**Total U.S. unproved**	**1536.38**
**Proved reserves**	**211.09**

**Total natural gas**	**1747.47**

Source: Energy Information Administration - Annual Energy Outlook 2009

Due to new drilling technologies, such as hydraulic fracturing technology [15] and horizontal drilling, that are unlocking substantial amounts of natural gas from shale rocks, the estimated gas reserves of the U.S. have surged by 35% in 2009. Shale gas is trapped underground in bubbles between thin layers of shale rock. The report by the Potential Gas Committee, the authority on gas supplies, shows the United States holds far larger reserves than previously thought. In fact, leading industry experts now believe that North America has more than 3,000 trillion cubic feet of proved natural gas reserves—enough to meet the current rate of U.S. consumption for more than 100 years [16]. This finding raises the possibility that natural gas could emerge as a critical “transition fuel” that could be used to mitigate the cost of shifting into a clean energy economy.

The single largest source of U.S. Natural Gas Supply is unconventional production, in particular natural gas in tight sand formations, which is predicted to accounts for 30% of total U.S. production by 2030. Production from shale formations however, is the fastest growing source.

## Supply implications on price

From the 1990s to the early 2000s, the price of natural gases has been stable and low compared to that of petroleum. This is because in the early 1980s (after the start of natural gas field price deregulation), the available supply exceeded the market demand, creating a “glut”. This is termed the “gas bubble”. The price at the wellhead for natural gas in 2002 was 22 % *less* in real terms than it was in 1985 [17]. The low price of natural gas has the effect of encouraging more demand, but provides a disincentive for gas producers to find more gas. As a result, the gas bubble eventually unraveled until supply and demand is approximately balanced. This means however, that an unexpected shock to the supply and demand dynamics of natural gas, in the form of a tsunami or a new technology, would lead to a rapid change in price. The discovery of hydraulic fracturing and horizontal drilling technologies in the 1995 by Barnett Shale of Texas represented such a shock.

The development of U.S. shale deposits began to be aggressive in 2005 when it was reported by the EIA that “technically recoverable” gas resources was 862 Tcf [18]. In the early part of 2011, natural gas prices are hovering around the lowest since 2002. April natural gas futures weighed in at about $4.17 for a million Btu, the amount of energy contained in about 8 gal of gasoline [19]. Of course, some of this could be attributed to the recession, which has decreased the demand for gas, but for the most part experts believe it is the result of the rising production from North American shale rock formation.

This has some behavioral implications for users of natural gas. For example, in February, trustees at Purdue University, home of the Boilermakers, voted to cancel a $53 million project to upgrade the Wade utility plant with clean coal technology. Instead, they plan to install a natural gas unit to replace the existing 50-year-old Boiler No. 1 [20].

Furthermore, given the uncertainty in the climate legislation of the U.S., natural gas appears more and more like a strategic choice. In the absence of a national cap-and-trade legislation, gas-fired plants are still cheaper, and require shorter-term commitments, than plants fired by other “clean tech” fuels. In fact, according to the most recent report done by Energy Information Administration (EIA), costs for combined cycle natural gas plants are expected to stay stable and affordable at or below $1,000/kilowatt, while the future capital cost for nuclear plants will reach nearly $5,300/kilowatt [21]. In the same report, EIA also predicts substantial increases in cost of coal and wind-powered plants. When it comes to generating electricity, natural gas is not only a cleaner but more cost-effective. Table 5, provided by the EIA, shows that natural gas generated electricity will have the lowest cost in 2016, compared to coal, wind, and solar.

**Table 5:** New generating technologies - 2016

Plant type	Capacity factor (%)	Total system levelized cost (¢ per KWH)
Natural gas - combined cycle	87	6.31
Natural gas - conventional	87	6.61
Natural gas - combined cycle with CCS	87	8.93
Coal - conventional	85	9.48
Coal - advanced	85	10.94
Coal - advanced with CCS	85	13.62
Wind - onshore	34	9.70
Wind - offshore	34	24.32
Solar - PV	25	21.07
Solar - thermal	18	31.18
Nuclear	90	11.39

*Note: “levelized cost” of electricity takes into account all the costs of power over its entire life, *including capital costs to build plants.*

*Source: Institute for Energy Research*, *using data from EIA Annual Energy Outlook 2011*

In the alternative scenario that a nationwide cap-and-trade legislation does pass, greenhouse gases will be priced to reflect their social costs, i.e. global warming, health consequences, ecological damages…etc., natural gas would also be cheaper than traditional, GHG-intensive fossil fuels. Investing in natural gas therefore, could lead to a win-win situation.

Of course, this is only a general analysis. An article that appeared in *Environmental Finance *[22] shows that, under certain assumptions and postulations of CO_2_ prices, coal-fired power plants could be a viable option for some firms. [See Table 6 for the calculations made in the article]

**Table 6:** Cost estimates for emission-neutral power plants ($/MWh)

Plant type	Levelised capital costs: over 20 yrs ($/MWh)	O&M costs (variable and fixed) ($/MWh)	Total fuel price ($/MWh)*	Total cost† ($/MWh) (CO_2_ = $5)	Rank 1 (CO_2_ = $0)	Rank 2 (CO_2_ = $5)	Rank 3‡ (CO_2_ = $10)
Wind (50MW)	19	10	-	29	2	1	1
Coal (400 MW)	9	7	11	34	1	2	2
Gas CC (400(MW)	4	4	27	37	3	3	2
Gas CT (80MW)	14	2	44	64	4	4	3
Solar (5MW)	77	3	-	80	5	5	4

*Coal price = $1.21/million BTU (about $25/short ton), gas price = $4/million BTU; † includes CO_2_, SO_2_, NOx costs (see Table 2); ‡The costs for opertaing a coal unit and a CC are approximately equal at $10/ton CO_2_

Source: the table is reproduced from the Environmental Finance source cited in the main text

Far from undermining our conclusions, what these calculations shows us is that natural gas is still a very competitive fuel in most cases. For these calculations are made under the assumption that technology is constant and that there are zero emissions associated with coal extraction, which is unrealistic.

## The US natural gas market: a closer look

The US has a vast network of high-pressure interstate pipelines or trunk lines that carry gas from the major supply areas - notably Mexican Gulf (onshore and offshore), the lower Midwest, the Permian Basin on the Texas/New Mexico border, the San Juan Basin in the southwest if the Rockies- to the main areas of consumption both within the producing regions and in the Northeast, Midwest of California. There are also a number of pipelines linking the principal producing fields in the Western Canada Sedimentary Basin with US markets in California, the Midwest and the Northeast (the Northeast being the largest consuming region). These networks are highly integrated so that gas from producing states in the Gulf region can in principle move just about anywhere in the system. The transmission system includes 270,000 miles of pipelines.

Nevertheless, the efficiency in U.S. natural gas pipelines does not guarantee the uniformity of natural gas prices across the nation. Instead, the price faced by each state/region is determined by regional supply and demand, its proximity to production (if it is further away from the production site, then prices would be higher to reflect transportation and storage costs), state-level regulatory environment, and the cost of natural gas that is flowing in the local distribution system. In the California Energy Crisis, for example, Californians faced higher prices of natural gas due to manipulations for Texas energy consortiums, which increased the cost of gas in the local distribution system.

In the past, the proximity to source of production is the most important factor. However, with the discovery of shale gas and the increased import of liquefied natural gas (LNG), the “geography” is slowly shifting. For example, states that are far away from production sources could invest in regasification terminals for LNG imports (obviously, they would only do so if the cost of building regasification plants is lower than that associated with transporting gas from the production source). However, there is still some inertia in this process as local gas infrastructures are already built to receive gas from far away, or have already purchased its gas supply under fixed-price long-term agreements.

The price of natural gas is not only different among states, but among type of users as well. For example, gas is cheapest for companies who purchase the gas in bulk as it flows from a well. This is known as the "wellhead price", which excludes the cost of processing and transportation. On the other hand, gas is most expensive for the homeowners because by the time the gas reaches the residential sector, it had already undergone an extensive distribution system, and incurred the fees associated with transportation, processing, metering, billing, distribution system maintenance, customer service…etc. There are also other types of natural gas prices other than wellhead, residential and Henry Hub prices, which are defined in Table 7[23].

**Table 7:** Different price quotes for natural gas

Natural gas consumer sectors and price quotes	
**City gate price** **	The price paid by a natural gas utility when it receives natural gas from a transmission pipeline. "City Gate" is used because the transmission pipeline often connects to the distribution system that supplies a city.

**Commercial price** **	The price paid by nonmanufacturing businesses engaged in the sale of goods or services such as hotels, restaurants, stores and service enterprises.

**Electric power price** **	The price paid by electric utilities and other companies who burn the gas to produce electricity.

**Henry Hub price**	Henry Hub is a pipeline terminal on the Louisiana Gulf Coast. It is the delivery point for natural gas futures contracts traded on the New York Mercantile Exchange. The "Henry Hub price" is the amount that will be paid for gas at the hub on a specified date in the future.

**Industrial price** **	The price paid by manufacturing companies who use gas for heat, power, or chemical feedstock. Includes those engaged in mineral extraction, forestry, agriculture and construction.

**Futures price**	A "futures price" is a quote for delivering a specified quantity of natural gas at a specified time and place in the future. Buyers who need a long-term supply at a known price will contract for gas with futures.

**Residential price** **	The price paid by homeowners who use the gas mainly for space and water heating.

**Wellhead price** **	The price paid at the mouth of a well for gas as it flows from the ground without any processing or transportation provided.

**FOOTNOTES**	** These are natural gas consumer sectors. Prices given for these sectors are averages. They are not fixed prices paid or charged across the sector.

Source: reproduced from Geology.com

For the purpose of the next section, we will focus solely on the Henry Hub prices. The next section is devoted to the study of two phenomena in the US natural gas market: the rise in price and the increase in price volatility. While they are closely related by the underlying causal factors, one need not imply the other.

## Price increase

**Figure 6:**
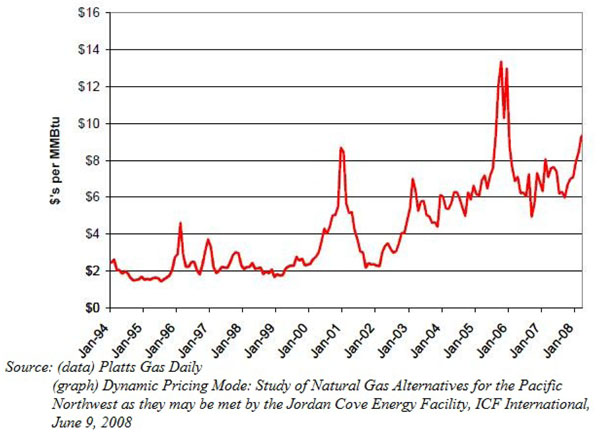
Recent historical trends for Henry Hub gas prices.

Throughout most of the 1990s, an excess of productive capacity in the U.S. created a "gas bubble" which allowed prices to remain relatively low and stable- $2 to $3 per MMBtu Since the early 2000s, the surplus of productive capacity had begun to unravel and we are left with a new price trend. Looking at the Henry Hub gas trends in [Figure 2], the North American natural gas market has undergone a fundamental shift that started in the beginning of the decade. Between 2000 and 2007, gas prices at most trading locations through North America averaged in excess of $5 per MMBtu. Since 2005, most averaged in excess of $6.50 per MMBtu [Figure 6]. The extent to which this price increase is felt by different sectors is difficult to quantify as we have mentioned earlier, the natural gas market is a segregated one with users facing significantly different prices.

There are a couple of reasons behind this persistent price increase, the most common one being the inflexibility/rigidity of natural gas supply and demand. This brings us back to our discussion about endogenous “features” of natural gas that determines its pricing structure. For one, gas production and transportation require large investment because of difficult geological conditions of extraction and production and transportation considerations. Also, once the project is planned out and its investment funds are committed, the project’s carrying capacity is usually fixed, rendering it difficult to keep up with changing demand trends. There is also the issue with time lags. In the US, when gas prices spike, producers increase their drilling but it usually takes around 18 months before such new drilling translates into additional production capacity. Part of this supply rigidity can also be explained if there are other countries involved in the “gas chain”. In such a case, price signals will not necessarily lead to investment, production, etc [24].

A lot of the price increase can also be attributed to expectations. Despite the abundance of shale gas, it is more expensive to produce than natural gas. Since it horizontal drilling is a relatively new technology, it needs to be supported by adequate human capital, which in turn represents an extra cost [25]. As for April 2011, Baker Hughes, one of the world’s largest oilfield services company, reports that of the 1782 rigs drilling in the U.S., 50% were drilling for gas, and of the 50%, only 57% will competent at drilling horizontal wells [26].

Some argue that the cost of finding, developing and producing shale gas makes it economically infeasible, given the current price of gas. According to Ben Dell of Bernstein Research in New York, gas prices have to be at least $8 per mcf to sustain shale gas production. Also it is argued that wells in one of the first shale field development with new technologies, the Barnett in Texas, have had faster-than-expected productivity declines. One large German bank's commodity group estimated the average economic life of shale fields is 10 years [27]. Since rock formations will collapse once significant amounts of gas have been extracted, industrial experts also predict that shale gas will become even more expensive to produce in the future. The expectation for higher prices for shale gas in the future could be reflected in current prices, which partially explains the persistent price increase.

Of course, there are other “exogenous” factors that contribute to this supply rigidity. The latter could be a result of supply restrictions, such as environmental pressures (occurring in 1990s to build power plants that are fueled by natural gas in order to economically comply with new source review regulations introduced in 1970s), difficulty in public land access, etc. Indeed, there are some analysts who argue that regulatory mandates are to blame for the price increases as they have prevented portfolio diversification of energy choices, which leads to markets that do not adapt to unanticipated and changing conditions.

**Figure 7:**
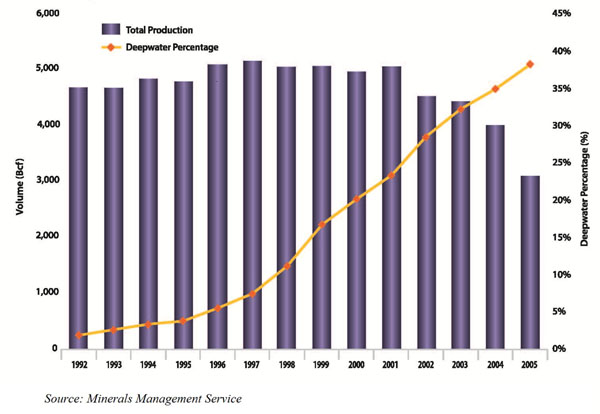
Natural gas production of the Gulf of Mexico federal offshore, 1992-2005.

Figure 7 shows the Gulf of Mexico production trends. As we can see, exogenous factors such natural calamities can be induce producers to cut their supplies (the three dips in production correspond to the three hurricanes). It should also be noted that due to the recent cold weather, a record amount of natural gas has been withdrawn from storage in February, dropping inventories below the five-year maximum for the first time in over a year [see section on Storage and Pricing]. Also, the high cost of replacing natural gas production across all basins has raised the price floor. This is worsened by the gradual reduction in supply from conventional gas basins and the steady increase from unconventional basins. Canada is also cutting its natural gas exports to the US due to the high domestic demand that it is facing. Demand rigidity on the other hand, comes from the inability of some end-consumers of natural gas to switch to a different fuel when gas prices are high [see demand responses].

Interestingly enough, high oil prices may also “allow” gas prices to soar due to competitive fuel switching. This is exacerbated by the fact that the petroleum supply industry tends to favor oil development over that of gas when prices are high, because oil development costs and the lead time to first production are usually less.

## Price volatility

The price of gas has always been volatile. A large part of this is due to its sensitivity to seasonality, as mentioned before. Since early 2008, however, natural gas prices hit the record volatility of 70%. There a few speculations as to why gas prices have become increasingly volatile. But most of them can be reduced to a tightening of supplies and a spiking in demand. The latter has been especially important in the past couple of years due to the sudden surge of “environmental conscience” all around the world, especially among the G20 nations. This is best captured by the agenda of the 2009 Copenhagen Climate Conference.

As price volatility is never desirable for any economic agent, it should be reduced through several means:

1) The regional price differentials of natural gas in the US generally reflect the cost of transportation from another region, improving the efficiency of transportation will also reduce the price volatility.

2) Local natural gas utilities could re-organize their portfolios to include more long-term fixed price contracts. According to American Gas Association, natural gas utilities tend to hold a larger percentage of long term “first of the month”, which adjusted monthly with the market. Instead they should invest more in stable long term contracts to ensure certainty and

3) Increased use of financial derivatives, such as futures, options and swaps, to hedge risk. These are contractual instruments that convey a right and/or obligation to buy or sell a commodity, like natural gas, at some item in the future for a specified price under specified terms and conditions. The transaction costs associated with such instruments are lower than that of physical hedges. The fact that these contracts are cleared through a central clearing system also guarantees the integrity and cost minimization of the transaction. The use of such financial derivatives are not common in the in the early 1990s, but grew dramatically from thereon. The number of natural gas contracts traded daily at the Chicago Mercantile Exchange (CME), the world's largest exchange operator, is 440,882 as of January 2011. This represents a 25% from the previous year [28]. In fact, it has been estimated that the value of trading that occurs on the financial market is 10 to 12 times greater than the value of physical natural gas trading. LNG swaps in particular, helps to move gas between regions, which would reduce volatility caused by rigidity in supply. They allow for short-term contracts and encourage speculative ventures and new entrants in the market. It should be noted however, that these hedging methods merely constrict the volatility of prices but do not guarantee the lowest price of natural gas possible. In order to achieve this, local natural gas utilities and their regulators should work cooperatively to better provide price stability for natural gas customers.

4) Improve the infrastructure of natural gas, which includes building more wells, pipelines and storage facilities. This would protect producers and consumers alike from unpredictable shortages in natural gas supply.

5) Some plants could also concentrate on building dual-fuel capacity, meaning that they could switch from one fuel to another at relatively little cost.

6) Although the North American natural gas market is still regional, new global developments in LNG transportation is paving the way for the emergence of a global gas market. In the past, technical challenges posed by transporting LNG, along with the environmental and safety concerns, have limited the imports of LNF in the U.S. to a mere 1% of total U.S. gas supply. Therefore, an expansion of LNG terminal import capacity and development of more efficient offshore regasification technologies is required to allow the U.S. to import more natural gas from abroad, which would stabilize price fluctuations due to tightness in supply. Similar investments could also be made for LNG cargoes and interregional pipelines.

In general, the prices of commodities with relatively constant supply and relatively variable demand tend to be more volatile. It can also be observed that the volatile spot prices of natural gas reflect the less developed, price dampening global trade of natural gas. Oil producers on the other hand, have unlimited access to world oil supplies so oil refiners in the US can easily smoothen out price spikes by importing oil from other countries.

An increase in the level of global trade may reduce price volatility of US natural gas. Although it is possible to import LNG from abroad (in fact we do see increased marginal supplies from abroad), it can also make US dependent on insecure foreign supply (like the case with oil), and is therefore a legitimate area of concern. While this is something that is often hotly debated by politicians and economists alike, the fact that natural gas reserves are more widely dispersed in the world than that of oil source is often overlooked. This is important as it lessens the danger of the US being dependent on a “sole source of energy”.

## Policy implications

It seems that in order to stabilize prices, the US natural gas market must increase the “safety valve” of natural gas supply. Whether this should be done by increasing domestic supply, or importing from foreign sources is something to be determined. There is of course, always the option of government intervention through the use or tax/subsidies and price caps. The case is weaker for the latter intervention due to California’s experience in the Western Energy Crisis mentioned above. Recently, the Obama Administration has also proposed to lower energy subsidies, especially in the oil industry, with the aim to lower greenhouse gas emissions. This may in fact increase the natural gas’ market share in the energy industry as oil prices rise.

Some analysts have also argued that the abundance of coal in the US (according to the EIA, the US still has 250 years of domestic supplies) justified the compensatory role of coal during the time tightening gas supplies. It should not noted however, that although existing coal fired plants are still cheaper for base load production than bringing new gas turbines, many coal plants will reach the end of their useful lives over the next two decades and gas turbines will have a strong advantage when new installations are being considered.

As a forecast for 2010, Bloomberg has predicted that natural gas prices in the US will begin to drop. Some analysts have shown that this depressing trend of natural gas prices does not have much to do with the increase in domestic output, which has remained resilient and dropped on a monthly basis since the last quarters of 2009. Instead, this downward trend seems to be caused by a decrease in domestic demand [29]. Of course, this does not mean that the US is using less natural gas now. On the contrary, they are using more natural gas than ever, and we predict that the volume of natural gas that will be used in the foreseeable future is even larger due to President Obama’s vow to cut U.S greenhouse gas emissions by 17% at the Copenhagen Climate Conference [30]. The root of this almost two-fold decrease in domestic gas demand then is caused by an increase in volume of LNG imports, mostly from Canada.

In the following section we look into some of the factors that affect the competitiveness of natural gas as a primary fuel. This includes, but is not limited to, cost of storage, transportation and distribution.

## Natural gas storage

Gas storage addresses short-term fluctuations in the market for natural gas, such as natural disasters and gas field malfunctions. Adequate storage serves as a buffer between transportation and distribution to ensure a constant supply of natural gas. Another important use of storage includes the leveling the cost of natural gas by controlling the supply and making fluctuations more predictable.

In order to measure the quality of storage, it is important to know the measurements of the storage space. The total storage capacity refers to how much natural gas the facility can hold until optimal conditions. Base gas must be kept in the facility at all times to maintain the pressure. The lowest volume of base gas required, the more economical the storage facility.

## Distribution of natural gas storages in the U.S

There are 120 gas storage operators in the U.S., with a combined working gas capacity (the total volume of natural gas that can be delivered to the market) of about 4.1 Tcf. However, the distribution of these operators is quite geographically uneven. Most operators are located in the northeastern side of the country and the gulf coast region. There are very few storage facilities located on the western coast and mountain regions. However, this distribution is explicable in several ways. The northeastern part of the country tends to experience greater fluctuations in temperatures and colder winters than the rest of the country; this fact indicates higher demand for storage in these regions. There are also an abundance of depleted, recyclable reservoirs in these regions that can be converted to gas storages. By contrast, the western United States enjoys warmer weather, and therefore lower demand for natural gas storage. The mountain regions are sparsely populated, which leads to less demand for natural gas. The gulf regions, though with similarly low demand, are big producers and exporters of natural gas, where storages are also needed to balance the market.

## Regulation and ownership of storages

There are about 80 corporate entities that operate the 400 + storages across the U.S. The entities are either subject to the regulation of the state they operate in, or subject to the jurisdiction of the Federal Energy Regulatory Commission (FERC).

Interstate and intrastate pipeline companies own most storages. These companies use storages to perform load balancing and supply management for their pipelines. They also lease their storages to others in the industry. However, interstate companies rarely serve the end users directly.

The Local Distribution Companies (LDC) directly serves the customers over certain regions. These companies are owned either by private investors or by the local governments. They control the flow of gas through the pipelines to individual households. Before 1992, natural gas storage was a product sold by pipelines to the LDC; it was heavily regulated to meet the transportation and distribution needs. After the market deregulation through FERC Order 636, storages become available to anyone for commercial purposes. In other words, after 1992, many storage facilities have become profitable businesses where the private owners inject gas when price is low and deliver it when the price is high. This deregulation gave greater flexibility to storage managements in recent years.

## Storage facilities

Although natural gas storages serve the same purposes, there are many methods to build them, with each having its advantages and disadvantages. Primarily, stored gas is held in underground formations such as depleted oil or gas reservoirs (the most common), in natural aquifers, or in cavities created in large underground salt deposits.

In the United States, gas is most commonly stored in depleted natural gas or oil fields near the center of consumption. The great advantage of the conversion field is its wide availability all around the country. These formations offer vast spaces that are geologically capable of holding natural gas in great volumes. In addition, the old reservoirs provide left over equipments and extraction networks that can be reused to save the cost of construction. These include existing wells, gathering systems, and pipeline connections. The conversion also helps recycle the old fields rather than abandoning them, which is an environmental benefit. In general, this type of facility is the cheapest to construct and maintain. However, some reservoirs require greater base gas volume and experience difficulties in injection and delivery. Also, depleted fields are rare in some rural areas.

Another common method is the conversion of aquifers found primarily in Midwestern United States. An aquifer may be easily available, however, only certain modified aquifers that has rock formation overlaid with an impermeable cap rock can be used for storage. There also needs to be extra monitoring effort on aquifers. Aquifers also take high base gas volume, at about 70%. Still, aquifers are better alternatives when there is an active water drive that enhances deliverability and when depleted reservoirs are not present.

A third method is the use of salt caverns. They are commonly found in the gulf of the United States. The cavern is created using fresh water to dissolve cavities in the salt formation, which forms large quantities of brine. Some great advantages of salt caverns are high withdrawal and injection rates and low base gas requirements, at around 25%. However, the construction of the cavern can be more expensive than depleted field conversions, especially in regions outside of the gulf coast. The brines are difficult to dispose and cause environmental damages.

Rarely, abandoned mines and hard-rock cavern are used to store natural gas. These facilities may cost less overall but are hard to find. The natural gas can also be stored in tanks as liquefied natural gas (LNG). LNG condenses the gas and allows it to become portable.

Gas is injected and withdrawn from these storages using the same type of well drilling and production equipment of natural gas fields. The capacity of storage, however, tends to decline rapidly among these storages. On average, gas storage wells in the United States lose about 5 percent of their ability to inject and withdraw gas each year. This problem is caused by the buildup of calcium carbonate and organic residue that clog the openings.

## The costs of storage

The construction or conversion of a natural gas facility can be extremely expensive. For example, the development cost of a salt cavern ranges from $10 to $25 millions per billion cubic feet of working gas capacity. The cost of construction escalates with the type of facility (salt cavern is the most expensive), the difficulty in locating and testing a storage site, the complicated geology of the site, the power needed to operate the facility, the distance from the consumption center, regulatory restriction, and various environmental issues.

The major cost in recycling depleted reservoirs and aquifers are the base gas injection, which account for more than 50% of the total capacity. The major costs in salt caverns are leaching and brine disposal, which are both expensive and polluting.

Other costs include the service costs to both deliver gas to and from maintain the facility. They can be as expensive as over $10 millions annually per facility. They include the cost of using interstate pipelines, tariffs, electricity, and storage services.

Lastly, there are always those per unit costs associated with injection of natural gas, its storage, its capacity depletion through leakages, and its extraction. This can cost up to millions of dollars, and vary based on the nature of the facilities.

## Why natural gas storage may be cheaper than oil and coal storages

The cost advantage of natural gas storage over that of other fuels can be best understood when one considers the risks involved. Oil spills causes extreme environmental damages especially to organism in water; in addition, it is nearly impossible to remove the spillage. The recent BP oil spill of 2010 is such an example. Not only did the BP suffer from the enormous clean-up cost, its “brand” is greatly compromised due to the immense ecological damage the spill had on the natural environment. On the other hand, leaked natural gas, though still somewhat polluting, vaporizes and disappears quickly in the atmosphere.

Similarly, coals contain the compound *marcasite* that risks spontaneous fire accidents when in storage. The fire can ignite a majority of coals in storage at once. It will then penetrate the surface and create devastating wildfire. This fire would release large amounts of CO_2_. By contrast, natural gas would not combust on its own.

Secondly, natural gas is stored in underground facilities that can hold a great volume at a time. Due to weight and volume, natural gas storage is cheaper than the storages of liquid and mineral in general. Whereas natural gas can be stored in a great variety of facilities, the means to store oil and coal are much restricted.

Of course, there is the recent Fukushima Nuclear Plant incident in Japan, from which one could infer the immense costs associating with handling nuclear wastes. In particular, the storage of fuel rods, the most radioactive of all nuclear wastes.

Crude oil is generally stored and imported in medium sized tanks and pipes. It cannot be sealed within underground formation, as it is a dissolvable liquid substance. Therefore, oil in storage is much restricted in volume. Additionally, oil is far more difficult to transport than natural gas, which is much lighter. It requires more labor for crude oil to be injected and pumped from the tanks than for natural gas to be supplied through pipelines.

Similarly, coal storage is more expensive than natural gas in that coals have to be manually stored into and delivered from the storage using carts or other vehicles. Also, we should store more gas than oil and coal because the unit price of natural gas is cheaper than the prices of the latter two. Such, the demand for natural gas is rising much faster than that of coal and oil. This means that in the near future people will start using more gas, whereby investments in the storage of the gas becomes increasingly economically rewarding.

## Ways to improve storage

Given the increase of natural gas demand, it is important to make natural gas storage more environmentally friendly and economically efficient. One existing proposal from the U.S. Department of Energy is to somehow to make the facility to chill the natural gas and reduce its volume, which reduces the burdens of constructing a large storage and increases the total capacity. This proposal would work extreme well on salt caverns that are very expensive to construct due to disposing of the leached brine; this would make salt caverns more affordable to areas outside of the gulf coast.

To chill, one can freeze the natural gas in the presence of water, and turn it into hydrates. The hydrates store natural gas in exceptionally large capacity. It is even proposed that an over 100 cubic feet of natural gas can be stored in one single cubic foot of hydrate.

Also, one can expand storages by trying to convert other formations into gas containers. This includes limestone, granite, and sandstone formations that are found at random locations across the country. These can become great substitutes for the locals wherever the three main formations are absent.

Lastly, we may further invest in privately owned, portable home natural gas tanks. We can liquidize the natural gas immediately from the gas field and store it into steel tanks. These containers can serve the users at anytime they want. They can take the tanks on vocations and use them wherever the access to gas is otherwise unavailable.

## Transportation and distribution costs of natural gas

The transportation of natural gas involves a network of interstate and intrastate pipelines that carry gas from the area of production to the end users. The network consists of three main systems: the gathering system, the interstate network and the distribution system. The gathering system is a set of low-pressure and low-diameter pipelines that transport raw natural gas to processing plants. The distribution system transports ready-to-use gas to local regions. The diameter of these pipes range from 6 to 48 inches. During distribution, gas travels at pressures from 200-1500 pounds per square inch. This can reduce the amount of gas being transported by up to 600 times. Compression stations are places at intervals of 40-100 miles along interstate pipelines in order to ensure that the gas remains pressurized, powered by small amounts of the gas being transported.

The low density of natural gas makes it difficult to transport and store. The natural gas pipelines are economical, but many in North America are close to reaching capacity. This leaves the politicians worrying about potential storages. Pipeline transport is not practical for overseas transport. It is preferred only for distances 2000km overseas and twice that over land. LNG carriers are used to carry LNG overseas and tank truckers are used to transport LNG and CNG (Compressed Natural Gas) over short distances. The transportation of LNG requires the building of liquefaction plants. This can be a capital-intensive process.

CNG is natural gas stored at pressure. The increased pressure allows large volumes of gas to be contained and transported within a given unit of space. It is necessary to compress natural gas for pipeline transport. The density of CNG can be reduced by refrigeration and this allows for greater transportation volume. Though compression and decompression equipments may be cheaper and more economical for smaller unit sizes, the transportation of CNG generally requires over 200 bars of pressure. The investment into and the operating costs of CNG carriers are the downside of CNG transportation.

## Transportation and distribution costs of oil and coal

The transportation of oil is a riskier, and therefore more expensive, undertaking than the transportation of natural gas. Much of U.S. oil (51% according to EIA) is imported and many of the oil tankers and pipelines flow through some of the most volatile regions of the world, a national security disruption or international conflict can have significant impacts on the price of the fuel. 40% of the world’s oil flows through thousands of miles of pipelines, often in hazardous areas, and a slight puncture to a pipeline can deem it non-operational. Because of the length of these pipelines, they are difficult to protect, and insurance premiums have been rising, and are thus just as vulnerable as tankers.

Maritime insurers have begun to raise rates for tankers in risky waters – for example, premiums for tankers passing through Yemeni waters tripled since the attack in Yemen. For a tanker with a cargo of two million oil barrels, the rate jumped from $150,000 to $450,000, consequently adding 15 cents to the final delivery cost of oil – and this only includes the insurance for the ship and not the cargo on board. The rise of terrorist attacks and other disruptions will increase the costs of protecting tankers, pipelines and oil terminals and be reflected in the final price of oil.

In addition, since more heavy oil is being pumped rather than lighter crude, the fact that heavier crude flows more slowly though pipelines reduces the volume being transported, making it more expensive to transport. To try to speed up the oil flow, oil companies sometimes attempt to dilute it or heat it, techniques which can be expensive and complex.

Below illustrates the breakdown of costs with every $1 spent on natural gas:

From wellhead to pipeline grid: $.30

From grid to processing plant: $.50

Processing and compression: $.90

Royalties: $.70

Operating costs: $.20

Interest on debt: $.50

*Finding*, *development and acquisition: $2.00*

*Source: “What is the breakeven price for Natural Gas Producers?” Keith Schaefer*, *Resource Investor: News that Trade*, *April 30 2009*

The above pricing information above is based on interviews with management from a Canadian natural gas company, but the process is very similar in the United States. These conservative pricing estimates show that the break-even for natural gas companies is near $5 for low-cost producers (for $3.40/mcf gas). Transport costs alone make up $0.80 of total costs, and this cost rises significantly with further distance between wellhead and grid and between grids to processing plant. Finding, development, and acquisition, often termed FD&A, also makes up a significant portion of costs, and this area in particular is in need of significant investment if drilling natural gas is to be an attractive potential investment.

The transport of natural gas is much cheaper since the primary transport method is via pipeline across domestic regions. The abundance of natural gas within the United States thus allows for cheaper transport costs of CNG within domestic borders, whereas the transport costs of oil are much higher and have high volatility due to costly and time-consuming transport across long distances via ships.

In a report published on April 2011, the U.S. Department of Energy provided the following breakdown estimate for each $1 spent on gasoline:

**Taxes**: $0.13

**Distribution and Marketing**: $0.8

**Refining**: $0.14

**Crude oil**: $0.65

The crude oil costs are largely determined by crude-oil suppliers, the Organization of the Petroleum Exporting Countries (OPEC) in particular. OPEC is composed of 13 countries, based largely in the Middle East, and is responsible for 40% of the world's oil production and holds the majority of the world's oil reserves. OPEC’s dominance means that they can engage in monopolistic practices that are detrimental to oil prices around the world. In April 2001 for example, OPEC reduced its collective production by one million barrels per day. This was at the same time that American consumers saw gas prices rise, hitting an average high of $1.71 per gallon on May 14, 2001.

The refining cost of crude oil depends on what type of crude oil it is. Crude oil can fall into a number of categories, and some crude oil is takes less effort to refine than others. One of the main costs of oil is the taxes. Taxes on gasoline vary between states, but are generally quite high because in addition to the federal and state governments excise taxes, there could state sales taxes, gross receipts taxes, oil inspection fees, underground storage tank fees and other miscellaneous environmental fees.

Table 8 summarizes the break-even estimates for oil in different oil-producing sites. It is clear that since the break-even points are much lower in the oil industry than in the natural gas industry, oil is currently a much more profitable investment area, and will continue to be so unless incentives for investment into natural gas are increased.

**Table 8:** Breakeven prices for oil

Oilfields/sources	Estimated production costs ($ 2008)
**Mideast/N. African oilfields**	6 - 28
**Other conventional oilfields**	6 - 39
**CO**_2 _**enhanced oil recovery**	30 - 80
**Deep/ultra-deep-water oilfields**	32 - 65
**Enhanced oil recovery**	32 - 82
**Arctic oilfields**	32 - 100
**Heavy oil/bitumen**	32 - 68
**Oil shales**	52 - 113
**Gas to liquids**	38 - 113
**Coal to liquids**	60 - 113

*Source: “Oil Price Predictions and Breakeven Prices”*, *Richard Shaw*, *Seeking Alpha*, *December 25 2007*

According to the International Energy Agency World Energy Outlook’s study done in 2008, regarding oil production costs by country, the operating and capital costs of oil range from $6/barrel to about $40/barrel, which are significantly higher than that of natural gas.

## Distribution of natural gas and the associated costs

Distribution of natural gas includes large industrial, commercial and electric generation customers as well as smaller individual users. While the larger consumers receive natural gas directly from interstate and intrastate pipelines, the smaller users receive it from local distribution companies (LDC), which typically take ownership of the gas once it is received and are located according to geographic regions to serve as delivery points to local users. LDC are either the property of local governments or investor-owned, and transport gas from delivery points along the larger interstate or intrastate pipeline system and into miles of small-diameter local distribution pipe. There approximately over 1,000,000 miles of distribution pipes in the United States. The delivery points to LCD are called “city gates”, and these serve as important points for determining the market price of natural gas, based on the supply and demand flow.

Due to the extensive infrastructure required in the delivery of natural gas across the country, the distribution costs account for a bulk of natural gas costs for smaller consumers who receive gas through LCD. The larger users of natural gas, such as industrial consumers, can take advantage of lower unit costs through the distribution of large volumes through wide-diameter pipes. Distribution companies face the expenses and inefficiencies of delivering small amounts of gas to small volume gas consumer in many different geographic locations. The EIA estimated that, the typical small volume consumer faces a natural gas bill, which is composed of up to 47% of distribution costs, while the physical commodity price comprises about 34% of the bill and the transmission and storage costs make the remaining 19%.

Traditionally, rigid steel pipes were used to construct distribution networks; however, new technology is allows the use of flexible plastic and corrugated stainless steel tubing instead of rigid steel pipe. These new types of tubing allow cost reduction and installation flexibility for the local distribution companies and the natural gas consumers.

Another innovation in the distribution of natural gas is the use of the electronic meter-reading systems. On site meters track the volume of natural gas consumed at a certain location by measuring the amount of natural gas consumed by any one customer. Meter-reading personnel had to be dispatched to record these volumes, in order to bill customers correctly, but the new electronic meter-reading systems are capable of transmitting this information directly to the local distribution company. These results in cost savings for the LDC, these savings are in turn passed along to customers. The installation of natural gas distribution pipe requires the excavation of trenches into which the pipe is laid, the same process as for larger pipelines. New trenching techniques allow the installation of distribution pipe with less impact on the above ground surroundings. The guided drilling systems are used to excavate an underground hole in which the pipe may be inserted, and this can lead to significant excavation and restoration savings. This is especially important in scenic rural environments and crowded urban settings, where the installation of natural gas distribution pipes can be a major inconvenience for residents and business owners.

Supervisory Control And Data Acquisition (SCADA) systems, are also used by local distribution companies; these are similar to those used by large pipeline companies. The SCADA systems can assimilate gas flow control and measurement with other accounting, billing, and contract systems that provide a comprehensive measurement and control system for the LDC. This allows accurate, timely information on the status of the distribution network that can be used by the LDC to ensure efficient and effective service at all times.

There are of course, other advantages of investing in natural gas that is not directly related to the cost reducing objective:

## Flexibility and capital investment

Natural gas electric generation plants can range in size whereas coal fired and nuclear powered plants are not as flexible and perform only large-scale generation. This forces them to produce more electricity to be economically efficient. Since the demand for electricity is expected to rise modestly in the future, electricity suppliers face the tough decision on whether or not they should make the large capital investment necessary for coal or nuclear power generating plants. Lower capital costs are required for natural gas-powered facilities, making it more practical to increase generational capacity as demand continues to grow.

Natural gas powered generation plants are operationally flexible and used to meet changing short-term peak demands. These plants can be turned on and off quickly and easily to respond to weather-related or other short-term demand fluctuations. Since neither coal nor nuclear generation plants demonstrate this flexibility, natural gas powered generators stand as an attractive operationally efficient option to manage the volatility of demand.

The limited capacity if interstate and intrastate pipeline systems in effect creates a ceiling on how much natural gas can be delivered to the market. There are currently 220,000 miles of pipelines in North America, but natural gas pipeline companies must invest in the expansion of this infrastructure to be able to meet project demands. So far, the industry has responded with relatively rapid expansion of infrastructure to keep up with growing supplies, discovery of new resources, and demand growth with 5,000 miles of pipelines completed in the past three years.

## Other long term investment in natural gas

Generally, there are several areas of investment in the natural gas industry. The investor can invest in the search and exploration of producing site; invest in the construction of the site; invest in the production of natural gas; invest in pipelines and transportation of natural gas; invest in the storage facilities and others.

One possible new area of investment is in fuel cells. The cells extract hydrogen from the gas and combine it with oxygen to produce water, electricity and heat. They are efficient and convert 60% of the energy in gas directly into electricity. These fuel cells become generators to power up cars and provide energy sources to households and factories. If this technology is further invested, natural gas powered fuel cells may lead to an energy system run on emission free, non-depleting hydrogen.

A second new area of investment is in gas made from biomass. The mass decays and produces methane overtime. We can invest in the capture of methane gas produced by landfill and sewage--this investment will not only provides a cheaper source for natural gas, but also prevents the spread of polluting methane.

It is important to recognize, however, that when it comes to energy issues, the politics are just as important as the economic issues, if not more important. In fact, it is usual the political forces, i.e. lobbyist, and not the economists, who determine the outcome of energy-related legislation.

## Natural gas political issues/regulation

The legacy of U.S. climate change legislation seems to be set by Title IV of the Clean Air Act Amendment (CAAA), also known as the Acid Rain Program due to its objective to reduce SO_2_ emissions (a major cause of acid rain) by 10 million tons from 1980 levels. What was notable about this program was the trading of emissions allowances, which would be issued, recorded and monitored by the EPA. The affected units can only emit SO_2_ only if they have enough permits to cover these emissions. Interestingly enough, despite Title IV’s resemblance to a cap-and-trade regime, the word [31], the word “trade” was never mentioned. The Acid Rain Program is favorable to natural gas demand as it led utilities and industries to use more natural gas in place of high-sulfur boiler fuels that contribute to acid rain.

Unfortunately, this benign legacy did not continue. On March 10, 2005, the EPA finalized the Clean Air Interstate Rule (CAIR) to reduce SO_2_ and NOx emissions through cap-and-trade, which is essentially an extension of the Acid Rain Program. However, a number of utilities, the State of Carolina and environmental groups filed a series of lawsuits against on the EPA and the CAIR. The case was argued in March 2008 and CAIR was vacated on July 11, 2008. The process also created an enormous amount of regulatory uncertainty for the public and utilities, dramatically limiting expenditures on emission reduction. AEP, an American electric utility, had to halt its project of carbon storage facility in West Virginia because of the uncertainty in the price of carbon [32]. In the absence of legislation, many utilities may also find if cost inefficient to switch to gas-fired plants.

However, the natural gas case has been gaining traction in Washington, D.C. as Oklahoma and Pennsylvania representatives Dan Boren and Tim Murphey have created a Congressional Natural Gas Caucus in an attempt to raise public awareness of natural gas. They noted the fact that natural gas is produced in 32 states and that the industry provides employment for approximately 3 million people in the United States. This data implies that 32 governors, 64 senators, and 324 Congress members from natural gas producing states may support efforts to boost the industry’s profile.

## Waxman-Markey bill

The American Clean Energy and Security Act of 2009 (ACES), also known as the Waxman-Markey Bill, was an energy bill in the 111^th^ United States Congress that aims to establish an emissions trading scheme for greenhouse gases, similar to that of the European Union Emissions Trading Scheme. The bill aims to create clean energy jobs, save consumer energy costs, increase America’s energy independence, and cut global warming pollution. The bill was approved by the House of Representatives on June 26, 2009 by a vote of 219-212, but died in the Senate.

One may be surprised to find out that some natural gas producers were actually opposed to this bill. The president of Independent Petroleum Association of America is quoted to claim that the bill "skews energy policy away from clean-burning natural gas. Second, it imposes new limits on gas and oil trading that will cripple independent producers' access to commodity markets.” [33] While it is true that the bill does not include any direct incentive programs for the natural gas industry, one would still argue that the passing of the bill would have benefitted any carbon friendly fuel by levying a price on carbon.

Furthermore, an energy expert at Rice University, Amy Myers Jaffe, pointed out how all the benefits to the coal industry provided by the Waxman-Markey bill undermine the global warming cause by freely allocating one third of the emission allowances to the coal-dominated power industry. Accordingly, natural gas would be more widely used than coal if it was not for the political boosts and benefits granted to the coal industry. From the point of view of economic efficiency, this argument seems to be flawed. Coase (2006) demonstrates that the allocation of allowances is independent of the efficient outcome, although it does have welfare implications. The zero cost of the allowances should not affect cost of cutting emissions and the resulting energy prices, i.e. if received a ticket to a concert for free, you would still sell it at the market price. Finally, the zero cost of the allowance may not necessarily deter firms from making pollution abating behavioral changes. It is the expected price of these allowances in the futures and not their current price that drives behavior.

## Boxer-Kerry climate bill

Barbara Boxer and John Kerry proposed the Boxer-Kerry climate bill. It is an ambitious plan to reduce carbon emissions by 20% by the year 2020. This plan includes strong incentives for the natural gas industry, including rewards for companies that switch from power sources with higher carbon emissions to those that emit less CO_2_, such as natural gas. Senator Mary Landrieu praised this bill saying that any move towards increasing natural gas use is a very smart thing to do and that leaders are beginning to learn more from different natural gas-producing regions of the country on the abundance of natural gas within our borders. She was one of nine senators who sent a letter to Barbara Boxer asking for more incentives for the natural gas industry. The producers of natural gas have also been aggressively increasing their lobbying efforts to ask for more natural gas incentives and have been gaining more support. Natural gas plants may be eligible for incentives as a fuel that produces half as much carbon dioxide as coal and a third less than oil. It can also work as a backup source for solar, wind and other renewable energy sources.

This bill however, has lost traction since the late 2009 and it seems unlikely that it would ever come to pass as a law.

## The Kerry-Graham-Lieberman climate bill

The Kerry, Graham, Lieberman climate bill includes titles covering a cross section for the nation’s top environmental and energy issues from expanding nuclear power and carbon capture and sequestration to revenue sharing for states that want to conduct more offshore oil and gas production. The trio proposes a reduction in U.S. greenhouse gas emissions by 17% from 2005 by the year 2020. This bill would strip the EPA of the power to regulate greenhouse gases. They are trying to encourage offshore drilling for oil and gas, increase U.S. nuclear-power plants and to ensure a future for coal. Though they support a “cap and trade” system, in which enables polluters to buy and sell credits to emit greenhouse gases, under an overall national cap, they prefer to call it the “market-based approach”. A fee would be placed on refined motor fuels, which the oil industry could pass on to the consumers; in this case the government would be blamed for the higher costs. The bill includes a border-protection mechanism that punishes developing countries with trade sanctions in an international climate agreement cannot be reached and then they do not do enough to curb their own greenhouse gas emissions. Overall the bill would set up new nationwide standards for energy efficiency and renewable energy, as well as ideas on carbon market regulation crafted by Sens, Maria Cantwell and Susan Collins. The bill dives into the subject of emission allowances; local distribution companies that service electric utilities and the natural gas consumers can expect free allowances through the year 2029. But the senators face a major issue over exactly what formula to use when giving the credits out to the power companies.

## Summary and conclusions

Given our research, we believe that there are several reasons for the United States to enhance the competitiveness of natural gas:

• Natural gas is a versatile fuel that can be used to power its residential heating, industrial, electrical generation, and transportation sectors for decades into the future.

• According to Seeking Alpha, The U.S. uses 25% of the worldwide oil supply and imports 65% of it. Natural gas is the only U.S. domestic fuel, besides from coal, that is abundant enough to reduce oil consumption over the next decade. The use of the vast US natural gas reserves and the nation’s 2.2 million mile natural gas pipeline grid are the best way to reduce foreign oil imports.

• Natural gas is environmentally friendly and is the cleanest (closest to being carbon neutral), and in most cases the most economically viable “transition fuel” to a “Clean Tech” economy. For example, NVG emit 20% less CO_2_ than gasoline powered internal combustion engines and no toxic particulates.

• Natural gas electrical generation is the preferred backup power supply for unreliable wind and solar energy. It is the ideal bridge to a renewable energy future.

• Natural gas electrical generators are more efficient and emit 50% less CO_2_ than the coal-fired plants. They do not emit any of the particulate matter or ash.

• The natural gas infrastructure can be used by the future hydrogen energy based economy.

• The “impending doom” of global warming has heightened legislative efforts for a comprehensive national climate change policy. Despite the inertia in congress to pass a national legislation addressing climate change, the proliferation of regional voluntary programs/efforts such as the RGGI, Californian AB ’32 [34], Chicago Climate Exchange [35], combined with competitive pressure on the international level (the success of the European Union Emissions Trading Scheme for example), will create an impetus for change. Investing in natural gas, therefore, is a strategic move.

Our research has also shown that there are some key barriers that prevent natural gas from becoming the prevalent fuel:

### Gas transportation

• Many utilities do not provide the necessary means for transporting natural gas from wellhead to gathering system. Also, owners of gathering systems often do not have the necessary incentives to maintain or expand existing systems.

### Infrastructure finance

• There are some major disincentives for investors to finance new gas infrastructure. Currently, disincentives include utility claims, which make the operation of a proprietary pipeline a violation and a “sham” that deprives the utility of rents.

• Unclear rules and regulations on the national level discourage investors to finance infrastructure projects.

### Regulatory clarity

• Oil and coal lobbies have been much more powerful and influential, as it was possible to fund more lobbying efforts since coal and oil are more profitable resources, with extremely high royalty costs and profit-making opportunities. This created a regulatory disadvantage for the natural gas industry, expansion of infrastructure, and investments in projects to expand use.

• There is no national-level policy regarding carbon in the U.S., such as a comprehensive cap-and-trade program, which makes agents hesitant to invest in natural gas.

• Currently, the United States Public Utility Commissions do not provide incentive for reducing emission of methane, which deters agents from switching from traditional fuels such as coal and oil to natural gas.

• The lack of legislation presented to effectively and significantly move the US away from gasoline powered automobiles makes it evident that President Obama and Energy Secretary Chu are not focusing on reducing foreign oil imports. The electric vehicle (EV) solution does not work over the short term because EV would have to be charged by coal-fired power plants.

• Lack of extensive technical knowledge and the existing opportunities regarding the natural gas market.

• In some cases, governments, through monopoly gas companies, could create a barrier to access this market.

• The network of long term supply contracts between gas producers and incumbent importers also makes it very difficult for new entrants to access gas on the upstream market.

The above suggests there are some areas that policy makers should focus on:

• Potential investors should be encouraged to finance new gas infrastructure. This will improve flows and remove bottlenecks.

• Incentives programs should be designed so that utility owners of gas-gathering facilities expand and maintain them to maximize the production of gas and it should be made clear that these facilities are different from those that cater to retail customers. The use of such facilities will help maximize the flow of domestic gas to the marketplace.

• If a utility is unable to receive gas, regulations should allow the producer to bring gas directly to storage facilities or through other pipelines to speed up the transport process and allow stranded gas into the marketplace.

• Clear rules must be communicated in order to allow potential natural gas infrastructure investors to fund projects without legal restrictions, challenges, or fees.

• LNG imports should be encouraged and more regasification plants should be built to reduce the tightness in supply (in fact, the U.S. had recently passed a legislation that would encourage more LNG imports from Canada).

• Given the Energy Secretary’s negative outlook of natural gas transportation, supporters cannot rely on the Obama administration for strategic energy policy. They need to take matters into their own hands and reach out to the American public with political activism and policy initiatives while pressuring automobile manufacturers to deliver NGV and refueling solutions.

• Similarly, agents should not wait for a national climate bill to pass before they take action in reducing their carbon footprint. Natural gas is less costly than other carbon neutral energy sources like nuclear and solar, and can be treated as a transition fuel into a clean energy future.

• An effort should be made to educate market participants about the natural gas market and the cost saving advantage of natural gas as a fuel.

• The importance of financial derivatives in hedging, price discovery and creating liquidity on the natural gas hubs should not be neglected. Market participants should be educated about the benefits of these instruments.

• The U.S. should draw lessons from examples abroad, such as the EU Commission’s efforts to stimulate competition between suppliers, the elimination of destination clauses on gas delivered to the EU, and the Second Gas Derivative.

It should be noted that the findings of this paper is preliminary at best, and more rigorous research is required to further quantify the benefits of natural gas.

## Competing interests

The team members have not had any financial or non-financial competing interest in relation to this manuscript.

## Supplementary Material

Additional file 1 Click here for file
