# Supplementary material for: The role of natural gas as a primary fuel in the near future, including comparisons of acquisition, transmission and waste handling costs of as with competitive alternatives
Source: Chem Cent J. 2012 Apr 23;6(Suppl 1):S4. doi: 10.1186/1752-153X-6-S1-S4 (PMC3332260; doi:10.1186/1752-153X-6-S1-S4)
Supplement: Additional file 1 [file 1752-153X-6-S1-S4-S1.pdf]

Appendix: Technology

New technology in Natural Gas

Distributed Power Generation

1) Microturbines

-Operates at hundreds of thousands of revolutions per minute at operating pressure of 60 psig (pound-force per square inch gauge)

Capacity: 30kW-300 kW

Installation cost: $1000

Mass production cost: $**400-$600**

Electrical efficiency: 30%

Advantages:

-Compact, light, and quiet without need for liquid cooling

-low nitrogen oxide combustion

-low maintenance required, making it attractive for on-site power generation for certain residential and commercial applications

Cost of power production: between 1/3rd-1/7th of local electricity prices

Companies producing these: *Allied Signal Power Systems, Rolls Royce-Allison Mobile Power Systems…etc*

2) Fuel Cells

Phosphoric Acid Fuel Cells

Cost: $**3000** per kilowatt

cost of PAFC must be reduced to about $1000-1500** per kW to compete with* ***0.05-0.06*** **per kW** hour of electricity

Electrical efficiency: 35%

***However, up to 80% thermal efficiency is possible if co-generation steam can be produced from the waste heat generated from the 200C° operation*

Commercial Uses: At present, total of about 170200kW PAFC units are used commercially in hospitals, nursing homes, office buildings, hotels, banks and a variety of power plants

<u>Molten Carbonate Fuel Cells (MCFC)</u>

Capacity: operate at 600C° and generally targeted for modular systems of 250kW capacity

Power production efficiency: 50%

Cost: installed cost needs to be reduced to $**1500-2000** to compete with electricity prices

* However advanced integrated MCFC co-generation systems could approach 70%

Major developers: *Energy Research Corporation, IHI Ishikawajima-Harima Heavy Industries Company Ltd. Hitachi*

<u>Solid Carbonate Fuel Cells</u>

This generates power electrochemically using natural gas, clean coal derived fuel gas, coal bed or coal mine methane, and landfill or waste water digestion gases. Unlike the molten one, the solid oxide concept uses zirconia ceramics, which must be operated at higher temperatures (1000C°)

Capacity: a unit generates 200kW electricity

Power production efficiency: 55%

*However, with cogeneration it could reach 80%

Installation cost: <$700

Advantages:

-SOFC can easily follow changing demands for electricity

-the solid state composition of SOFC allows many of the robotic mass production techniques that have reduced costs in the electronics industry to be applied to the power sector

Disadvantage: requires costly super alloys and exotic ceramics, gas leakage cracking and problems associated with high temperatures

Polymer Electrolyte Membrane Fuel Cells (PEMFC)

With this technology, natural gas is reformed with steam and/or by partial oxidation in a fuel processor and the resulting synthesis gas is further processed to produce a hydrogen-rich gas which is fed as fuel to the PEMFC

-operates at the lowest temperature (60-80C^{o})

Efficiency: 20%

Cost of production: Need to be reduced to $**200 per** kW (it needs to be **below 0.05 per kW hour** to be competitive)

Advantages:

-suitable for both stationary and propulsion applications

-PEMFC stacks with 3-5kW can target residential applications, while modular systems with 100s kW can target multi-floor apartment complexes and less than 100kW for small commercial business

Development: *Daimler-Chrsler, Siemens, NuPower, Mosaic Energy*

Innovative Natural Gas Usage

These examples are supposed to demonstrate how flexible natural gas can be

-Harley-Davidson Motor Company uses natural gas fired regenerative thermal oxidizer to destroy the organic compounds released from painting operations

-The California Medical Center generates electricity for most of the facility from two 800kWe engines. Waste heat from gas engines used to supply hot water, operate absorption chillers and sterilize medical supplies and equipment. But co-generation system isolates system from grid. Surplus power was put on the grid to generate revenue for the medical center

-Brach and Brock (fourth largest candy maker in Chicago) installed a Kathapac 1600 FV desiccant dehumidifier to deliver air at 15-35% humidity to pans of candy in order to ensure interrupted production of candies

Other developments…

Developments at IGT (Institute of Gas Technology)

-forced internal recirculation (FIR)

-METHANE de-NOx

-Oxygen-enriched air staged natural gas combustion→this will help gas industry meet stringent air quality regulations

-MORPHYSORB (new way of 'refining' gas to pipeline quality)→ reduced capital cost by 20%, operating cost 20%-60%

Please visit their website for more information:

http://www.gastechnology.org/webroot/app/xn/xd.aspx?it=enweb&xd=gtihome.xml

Appendix: Deregulation of Natural Gas Markets

Before Mandatory Open Access	After Restructuring
**At Wellhead**	
Producers contracted to sell gas to pipelines	Producers contract to sell gas to:<br>-End users<br>-LDC<br>-Marketers, including pipeline affiliates who sell to end users and LDC
Prices regulated by FERC	No price controls
Pipeline companies aggregated supply for customers	Customers aggregate supplies on contract with producers or marketers for this service
Pipelines responsible for supply reliability	Customers responsible for supply reliability
**Downstream**	
Gas customers obtained gas from pipeline companies through bundled sales and transportation service	Customers contract separately for gas from any seller and transportation from pipeline companies. Customers can buy a bundled service from marketers.
Transportation typically along one path, often involving a single pipeline company. Interconnections used mainly for emergencies.	Customers determine the least-cost combination of transportation route and source of gas supply
Operational adjustments to maintain system integrity handled entirely by pipeline companies	Customers are liable for penalties if they do not meet scheduled volumes and match receipts and deliveries within tolerances. Services available to avoid or reduce penalties
Pipelines companies controlled most storage for seasonal load balancing and operation control	Customers are responsible for reserving adequate storage to meet peak day requirements
Pipeline companies offered interruptible service when capacity not fully utilized	Firm shippers can release excess pipeline capacity and recoup oar tot all of reservations costs

http://www.wikinvest.com/image/Electricity_Source_Breakdown_2008.bmp

Appendix: Electric Generation

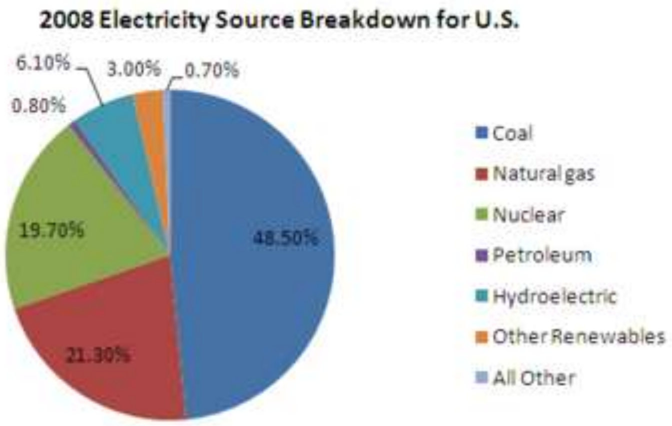

Sources: "Uses of Natural Gas", EIA, 2008

Energy Return by Source in 2008[22]	
	**Energy return on Energy Invested**
Coal-fired power plant	2.5
Nuclear power	4.5
Hydroelectric power	10
Wind power	35
Natural gas	10.3

Source: Suzlon FY 07-08 Annual Report, Management Discussion and Analysis, p. 5

Power Generation Costs for Various Energy Sources in 2008[21]

	Fixed Cost (cents/kWh)	Variable Cost (cents/kWh)	Total Cost (cents/kWh)
Coal	4.1	3.3	7.4
Natural gas	2.8	7.8	10.6
Nuclear	8.0	0.8	8.8
Wind	8.2	0.0	8.2

Source: "Cost of Wind vs. Fossil Fuels, The New Energy Coalition's Energy Matters", Vol. 5, No. 2 - July 22, 2008

Table 14. Estimated Annualized Cost of Power with Carbon Controls
(2008 $)

Technology (1)	Developer Type (2)	Non-Fuel O&M Cost (3)	Fuel Cost (4)	SO_2 and NOx Allowance Cost (5)	CO_2 Allow. Cost (6)	Prod. Tax Credit (7)	Total Operating Costs (8)	Capital Return (9)	Total Annualized $/Mwh (10)
**Coal Technologies**									
Coal: Pulverized	IOU	$5.57	$11.13	$0.61	$33.80	$0.00	$51.11	$49.58	**$100.69**
Coal: Pulverized/CCS	IOU	$13.48	$14.13	$0.77	$4.29	$0.00	$32.67	$78.87	**$111.54**
Coal: IGCC	IOU	$5.46	$10.41	$0.10	$31.61	$0.00	$47.58	$67.02	**$114.60**
Coal: IGCC/CCS	IOU	$7.10	$12.61	$0.13	$3.83	$0.00	$23.67	$95.25	**$118.92**
**Natural Gas Technologies**									
NG: Combined Cycle	IPP	$2.57	$30.57	$0.14	$13.06	$0.00	$46.34	$30.88	**$77.21**
NG: Combined Cycle/CCS	IOU	$3.68	$38.32	$0.17	$1.64	$0.00	$43.81	$51.09	**$94.90**
**Zero Carbon Technologies**									
Geothermal	IPP	$13.69	$0.00	$0.00	$0.00	$0.00	$13.69	$45.54	**$59.23**
Nuclear	IOU	$6.13	$5.29	$0.00	$0.00	($3.18)	$8.23	$74.99	**$83.22**
Wind	IPP	$6.67	$0.00	$0.00	$0.00	$0.00	$6.67	$74.07	**$80.74**
Solar: Thermal	IPP	$13.71	$0.00	$0.00	$0.00	$0.00	$13.71	$86.61	**$100.32**
Solar: Photovoltaic	IPP	$4.17	$0.00	$0.00	$0.00	$0.00	$4.17	$251.24	**$255.41**

Source: CRS estimates.

Note: Projections are subject to a high degree of uncertainty. These results should be interpreted as indicative given the projection assumptions rather than as definitive estimates of future outcomes. Mwh = megawatt-hour; IGCC = integrated gasification combined cycle; NG = natural gas; CCS = carbon capture and sequestration; SO_2 = sulfur dioxide; NOx = nitrogen oxides; O&M = operations and maintenance; IOU = investor owned utility; IPP = independent power producer.

Source: "CRS Report for Congress- Power Plants Characteristics and Costs", Stan Kaplan Specialist in Energy and Environmental Policy, Resources, Science, and Industry Division, November 13, 2008, Table 14
